# The role of feedback in emergency ambulance services: a qualitative interview study

**DOI:** 10.1186/s12913-022-07676-1

**Published:** 2022-03-03

**Authors:** Caitlin Wilson, Anne-Marie Howell, Gillian Janes, Jonathan Benn

**Affiliations:** 1 School of Psychology, University of Leeds, Leeds, United Kingdom; 2 North West Ambulance Service NHS Trust, Bolton, United Kingdom; 3 Faculty of Health, Psychology and Social Care, Manchester Metropolitan University, Manchester, United Kingdom; 4 Bradford Institute for Health Research, Bradford Teaching Hospitals NHS Foundation Trust, Bradford, United Kingdom

**Keywords:** Feedback, Feedback-seeking behavior, Prehospital care, Emergency medical services, Qualitative, Professional development, Staff wellbeing

## Abstract

**Background:**

Several international studies suggest that the feedback that emergency ambulance service (EMS) personnel receive on the care they have delivered lacks structure, relevance, credibility and routine implementation. Feedback in this context can relate to performance or patient outcomes, can come from a variety of sources and can be sought or imposed. Evidence from health services research and implementation science, suggests that feedback can change professional behavior, improve clinical outcomes and positively influence staff mental health. The current study aimed to explore the experience of EMS professionals regarding current feedback provision and their views on how feedback impacts on patient care, patient safety and staff wellbeing.

**Methods:**

This qualitative study was conducted as part of a wider study of work-related wellbeing in EMS professionals. We used purposive sampling to select 24 frontline EMS professionals from one ambulance service in the United Kingdom and conducted semi-structured interviews. The data was analyzed in iterative cycles of inductive and deductive reasoning using Abductive Thematic Network Analysis. The analysis was informed by psychological theory, as well as models from the wider feedback effectiveness and feedback-seeking behavior literature.

**Results:**

Participants viewed current feedback provision as inadequate and consistently expressed a desire for increased feedback. Reported types of prehospital feedback included patient outcome feedback, patient-experience feedback, peer-to-peer feedback, performance feedback, feedforward: on-scene advice, debriefing and investigations and coroners’ reports. Participants raised concerns that inadequate feedback could negatively impact on patient safety by preventing learning from mistakes. Enhancing feedback provision was thought to improve patient care and staff wellbeing by supporting personal and professional development.

**Conclusions:**

In line with previous research in this area, this study highlights EMS professionals’ strong desire for feedback. The study advances the literature by suggesting a typology of prehospital feedback and presenting a unique insight into the motives for feedback-seeking using psychological theory. A logic model for prehospital feedback interventions was developed to inform future research and development into prehospital feedback.

**Supplementary Information:**

The online version contains supplementary material available at 10.1186/s12913-022-07676-1.

## Background

The work of emergency medical services (EMS) has traditionally involved rapid transportation of patients to the nearest Emergency Department (ED). However, over the last two decades EMS have undergone significant changes signposted by landmark reports such as ‘Taking Healthcare to the Patient’ in the United Kingdom (UK) and ‘Emergency Medical Services: At the Crossroads’ in the United States [1–3]. EMS professionals now routinely assess and treat patients at home, refer them via alternative community pathways or bypass local hospitals in favor of advanced treatment in specialized centers. The resulting detailed assessments, complex decisions and lengthier transports [4, 5] have led to EMS professionals feeling more involved in patient care and wanting to know whether their clinical decisions were correct, known as “clinical curiosity” ( [6], p. 100).

There are, however, limitations in the current provision of feedback for EMS professionals. A study from Canada suggests that current prehospital feedback provision lacks structure, relevance, credibility and routine implementation [7]. A large-scale United States survey revealed that 45.5% of EMS professionals had not received feedback on the medical care they provided in a 30 day period [8]. Indeed, when encountering particularly difficult or unique cases, EMS professionals informally follow-up patients by contacting ED staff due to a lack of formal patient outcome feedback [9]. This is referred to by Croskerry as “specialty follow-up deficiency” ( [10], p. 1233) in relation to ED physicians.

The term “feedback” has many uses both within and beyond clinical practice and the academic literature on the topic. Feedback in the prehospital context may relate to performance or patient outcomes, can come from a variety of sources and can be either sought or imposed. In the medical education literature, evidence synthesis suggests that effective feedback should focus on the task rather than the individual and should be both specific and linked to personal development goals [11]. Within the broader implementation science literature, audit and feedback of individual performance is a well-researched phenomenon. Systematic reviews suggest that providing feedback to healthcare professionals results in small to moderate improvements in patient care and positive influences on staff engagement and mental health [12, 13]. This is especially relevant, in light of mental health disorders being more prevalent amongst EMS professionals than in the general population [14].

Health service researchers further suggest that feedback could improve patient safety [15–17]. This is highly pertinent because the uncontrolled prehospital environment represents an area of high risk for errors and harm [18]. A recent scoping review suggests that prehospital patient safety needs to become a more prominent consideration [19]. In areas such as non-conveyance, treatment, diagnosis and handover to hospital, the literature suggests this could be addressed by prehospital feedback interventions [18–21]. However, whilst research into feedback in certain areas of healthcare, e.g. primary care or outpatient settings, is well established, very little research addresses feedback in a prehospital setting.

Building on existing work in the field of audit and feedback, Brown et al. developed Clinical Performance Feedback Intervention Theory (CP-FIT) [22]. This cyclical model illustrates how feedback works within healthcare and outlines three factors that influence feedback effectiveness: feedback variables, recipient variables and context variables [22]. Brown et al. propose that these variables operate via a set of explanatory mechanisms; thereby, advancing our currently limited understanding of the mechanisms by which feedback influences outcomes in a real-world clinical setting [22, 23].

Alongside the feedback effectiveness literature centered on audit and feedback, a separate body of work has focused on Feedback-Seeking Behavior (FSB), which seeks to explore recipients’ motives for seeking feedback [24–26]. To fully understand feedback and its effects, there have been calls to integrate the two separate literature streams of feedback effectiveness and FSB [27].

Recent research into feedback provided to ED physicians identified the need to know whether recipients value feedback, highlighted the effects of feedback on personal and professional development, and suggested that further research is needed to determine the most effective feedback strategies and components in the ED environment [28–30]. These research gaps are echoed within the limited research on prehospital feedback, where studies by Morrison et al. and Cash et al. emphasize that further research is needed to explore feedback content and impact on prehospital practice [7, 8]. A recent, small-scale qualitative study suggests the lack of prehospital feedback reported internationally exists in the UK [31].

A deeper understanding of prehospital feedback is timely in light of several major health systems beginning to develop EMS guidance and policy relating to the provision of prehospital feedback. Examples from the United States are the recently published position statement by the National EMS Management Association [32] and the ‘EMS Agenda 2050’ report [33], which envisions EMS clinicians and systems receiving rapid feedback on patient outcomes to improve performance measurement, quality improvement and education.

The aim of the present study was to explore the perceptions of EMS professionals regarding current provision of prehospital feedback and their views on how feedback impacts patient care, patient safety and staff wellbeing. The latter included participants’ ideas concerning what could be achieved through enhancing existing prehospital feedback provision. The interview data was collected as part of a wider exploratory study of work-related wellbeing in EMS professionals in the UK, which included a prehospital feedback focus.

## Methods

### Study design and setting

This study employed a qualitative approach using semi-structured interviews with frontline EMS professionals from a single UK ambulance service. The research team consisted of qualitative researchers (BK, GJ, JB), a paramedic early career researcher (CW) and two undergraduate psychology students (FT, AH). The interviews took place via telephone or face-to-face in an ambulance service headquarters setting with only the participant and researcher present. They were conducted between April–June 2019 by three female members of the research team, who were from different backgrounds (health services research, psychological science). All interviewers were trained in qualitative methods with the senior researcher (GJ) being an experienced qualitative researcher. The study was carried out in accordance with the UK Policy Framework for Health and Social Care Research [34]. It was approved by the Health Research Authority (IRAS project ID 255406) and the University of Leeds ethics committee (PSC-518 22/11/2018). The Consolidated Criteria for Reporting Qualitative Research was used to guide study reporting [35].

### Selection of participants

Recruitment was conducted using purposive sampling and targeted the full spectrum of EMS personnel who had both patient contact and clinical responsibility (e.g. paramedics, emergency medical technicians [EMTs], clinical supervisors). Recruitment was conducted separate from the study team by the research department of the participating ambulance service through advertisement in the weekly ambulance staff bulletin. Recruitment reminders targeted specific staff subgroups with fewer responses to the first interview invitation. Staff expressing interest in participating were invited to email the research team directly, who screened participants for eligibility and provided a study information pack. Interviewers did not have an established relationship with participants prior to the study. To encourage participation, interviewees received a £25 voucher. Although patients were not interviewed as study participants, the research team involved patients through broader consultation with a patient-representative group.

### Data collection

Interviews were conducted using a semi-structured interview guide (Additional file 1), which was developed by the research team in consultation with patient representatives and reviewed by the ambulance service research department prior to study onset. The interviews sought to explore participants’ views on five different areas: work-related wellbeing, engagement, existing support systems, patient safety and feedback. Questions on feedback were informed by the existing prehospital feedback literature [7–9, 31] and stakeholder engagement with the ambulance service. This suggested that EMS personnel informally followed up on patient outcomes and therefore this type of feedback was specifically probed during the interview if it was not spontaneously mentioned by the participant.

Semi-structured interviews were chosen as they allowed the interviewer to guide the interviewee to address particular research questions whilst also providing flexibility for the discussion to move with the direction of the participant [36]. Prior to interview, respondents were provided with standardised research information regarding the study’s purpose and informed written consent was obtained. The interviews were audio-recorded and professionally transcribed. All data were anonymised to conceal participant identities. Interview transcripts were imported into NVivo (Version 12 Plus, QSR International) to support analysis.

### Data analysis

Data was analysed following Abductive Thematic Network Analysis [37], which builds upon the principles of thematic analysis and thematic networks [38–40]. Abductive reasoning was considered the most appropriate analysis approach for this study, because neither inductive nor deductive reasoning in isolation adequately addressed the study aim and intention. Abductive analysis seeks to construct new theory using iterative cycles of analytic interpretation, moving between the literature and empirical data [41, 42].

Themes were developed using iterative cycles of inductive and deductive reasoning, which involved drawing upon suitable theories from the feedback effectiveness and FSB literature [22, 24–26]. Developed themes were descriptive and conceptual in nature with linkages between themes depicted in a thematic map. The entire dataset was coded by a researcher from a paramedic background (CW) with input from an undergraduate psychology student (AH) and a senior health services researcher (JB). Although only one part of the interview guide specifically explored feedback, the interview transcripts were analysed in their entirety as part of the present study. Trustworthiness of the analysis was ensured through researcher reflexivity and an audit trail of theme development. The initial interpretation of the findings was presented to ambulance service representatives to ensure face validity.

Within certain branches of qualitative research, the concept of data saturation is firmly embedded, but Braun and Clarke [43] have challenged its relevance in reflexive thematic analysis. Their concerns similarly apply to the Abductive Thematic Network Analysis employed in this study, as the way findings were generated through abductive interpretation of the data brings with it an element of uncertainty and further interpretation of the existing or additional data may alter the developed theory [41]. In line with Braun and Clarke’s [43] suggestion to view ‘saturation’ as “an interpretative judgement related to the purpose and goals of the analysis” (p.10), we believe theoretical sufficiency was achieved through in-depth analysis in accordance with the study aim, as presented in the following.

## Results

Twenty-six participants were interviewed, with one interview recording error and one participant not covering the topic of feedback due to time constraints, resulting in a total of 24 interviews for inclusion in the analysis. The interviews lasted between 23 and 87 min, with a mean interview duration of 53 min. The participants were paramedics (*n* = 9), EMTs (*n* = 4), Emergency Care Assistants (ECA, *n* = 4), specialist paramedics (*n* = 3) and clinical supervisors (*n* = 4). The majority of participants were male (62.5%) and mean participant length in service was 9.6 years with a range of 1–29 years.

**Fig. 1:**
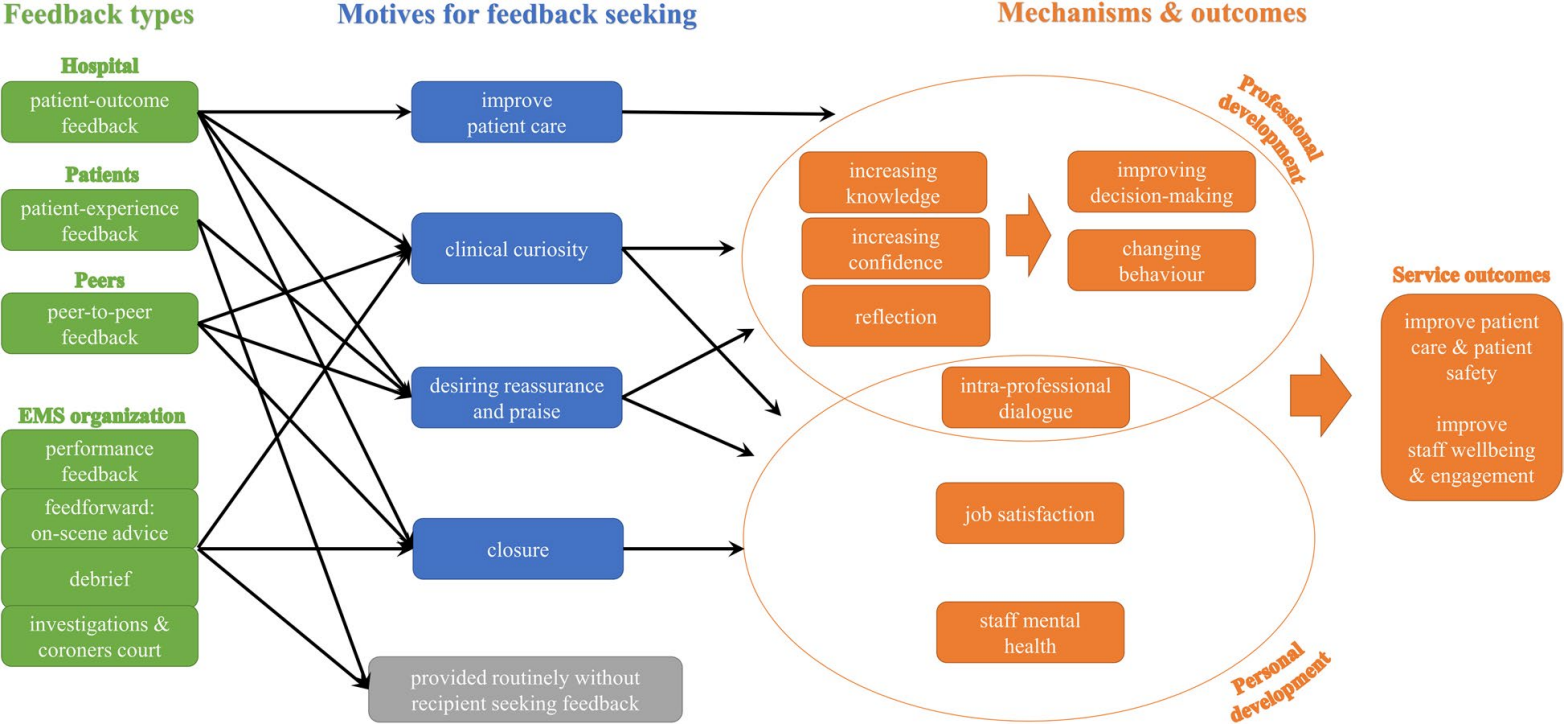
Prehospital feedback - thematic network map

During data analysis, four organizing themes were developed (feedback provision, feedback types, motives for seeking feedback, and feedback mechanisms/outcomes) each comprising a number of basic themes. To provide a visual summary, organizing and basic themes were captured in a thematic network map displayed in Fig. 1, with arrows indicating between-theme relationships.

The descriptive themes of feedback provision and feedback types are presented below, followed by the conceptual themes of motives for seeking feedback and feedback mechanisms/outcomes. Representative quotes are presented in tables for descriptive themes and integrated within the narrative text for conceptual themes, with additional illustrative quotes provided in Additional file 2. Our definition of mechanisms mirrors that of CP-FIT, i.e. explanatory mechanisms by which feedback impacts upon practice, not the actual mode of delivery of the feedback, which we have included under ‘feedback provision’ [22].

### Feedback provision

A majority of participants indicated that current feedback provision was inadequate, with feedback having to be self-initiated and some feeling like they only received negative feedback (Table 1). Participants consistently expressed a desire for more feedback, with several stating they wanted feedback, even if the message was not positive, for example: *“sometimes I think the not knowing is kind of worse”* (ECA, 3916). Three participants described their current feedback provision to be good, of which two were specialist paramedics who identified that the level of feedback they received was due to their specialist role. The remaining participant was a newly qualified paramedic who acknowledged that they received increased support due to being newly qualified. One male paramedic (P3924) suggested that females were more likely than males to seek and receive feedback but this was not mentioned by other participants so may be a unique perspective in our dataset.

**Table 1:** Feedback provision – basic themes and representative quotes

Theme	Representative quotes
Current feedback provision	“If you want to get feedback on a patient, you sort of have to go yourself and ask a doctor or ask a nurse about them” (Paramedic, P3912) “Feedback is only given if it’s negative.” (EMT, P3925)
Desire for feedback	“It would be nice to know what happens with patients sometimes because when we drop them off at hospital, we don’t often know what happens to them.” (Paramedic, P3919)
Barriers to feedback	“I think there’s probably some issues about information governance there and patient confidentiality” (Paramedic, P3912)
Feedback characteristics	“I think it’s the more challenging jobs I would want feedback on. If it’s our day to day jobs then I don’t need feedback, but the more challenging ones definitely.” (Paramedic, P3932)
Mode of delivery	“I think that for some things it would be good to have a face-to-face discussion with a more senior clinician but on a day-to-day basis it could be done digitally via email.” (ECA, P3912)
Antecedents	“I think feedback is a positive thing. I think most of us in the ambulance service have quite thick skins so we are quite good at taking criticism. Obviously, if there is something we have done wrong or there is something we think we could have done better, I think we are quite good at being able to say well ‘will you tell me how I can improve’ rather than being against feedback” (ECA, P3916)

Participants identified several potential barriers to prehospital feedback such as patient confidentiality, the time and effort involved in providing feedback, a geographically dispersed workforce, solitary working and a lack of data linkage. Participants wanted feedback to be detailed, timely, target driven, consistent and provided for ‘the right cases’. However, descriptions of what ‘the right cases’ were varied from difficult diagnoses, to non-conveyance, major incidents or critical patients. Participants described that they would prefer to receive feedback verbally as part of a face-to-face conversation, but acknowledged that written feedback via email or text may be more practical to provide. Participants reported being accepting of negative feedback so long as this was provided in a sensitive manner to avoid feeling like feedback was *“disciplinary, rather than a learning experience”* (Paramedic, P3912).

### Feedback types

As previously highlighted, the interview guide (Additional file 1) specifically set out to explore patient outcome feedback. However, participants referred to a wide variety of feedback types in their responses when talking about current and desired feedback despite not being prompted for them. Interpretation of the data suggests seven different types of prehospital feedback with four different sources (Table 2). Patient outcome feedback (predominantly, its absence) was most frequently mentioned: *“A lot of the time there is not much follow-up from patients”* (Specialist paramedic, P3904). Patient-experience feedback was the second most common feedback type mentioned by participants and was usually referred to in the context of thank you letters (and less frequently, complaints). Peer-to-peer feedback was described as feedback from *“your own crew mate that says: ‘You did really good on that job’”* (ECA, P3910).

**Table 2:** Types of prehospital feedback – basic themes and representative quotes

Theme	Representative quotes
Patient outcome feedback (Hospital)	“Sometimes I ask at the hospital if they can look up and see if the patient is alright. I don’t know whether we are supposed to but sometimes, you get quite close to patients and you want to know what has happened. It is a big frustrating part of the job.” (Paramedic, P3906)
Patient-experience feedback (Patients)	“It’s nice when we occasionally get patient thank yous. They get mailed to us, so you open all your boring post that you get at home and then you open this thank you letter. And that’s really sweet, it just lifts your day.” (ECA, P3910)
Peer-to-peer feedback (Peers)	“The nicest thing is if a member of staff after I have helped them out, says ‘Thanks for today’. That is the nicest thing because support and gratitude from your peers is one of the best feelings.” (Clinical supervisor, P3907)
Performance feedback (EMS organization)	“Formal feedback – we get our clinical supervisors out with us several times in the year so they can come and observe you and see how you’re doing.” (Paramedic, P3932)
Feedforward: On-scene advice (EMS organization)	“Sometimes you might be alone and you’re not sure what the best treatment is for this patient. You can always ring the clinical hub and they can give you some advice.” (Paramedic, P3905)
Debriefings (EMS organization)	“If you have been on a serious trauma job, there will be multiple team members there and you will do a debrief after the job. So, you will get feedback on how you did and that is really good moving forward or new things to learn like what you could have done differently, what you could have done better or what you think went well.” (ECA, P3916)
Investigations & coroners court (EMS organization)	“Within the incident reporting system you record a near miss and when it gets investigated you will get an e-mail back from the investigator about what they have found.” (Specialist paramedic, P3904) “Some of the big jobs that the police are involved with and you go off to coroner’s court, you might get a little bit of feedback from there.” (EMT, P3926)

Performance feedback related to regular appraisals or development reviews, whilst on-scene advice was captured as a feedforward mechanism, in which participants suggested they could *“ring the clinical hub and they can give you some advice”* (Paramedic, P3905). On-scene advice is conceptually different than the other feedback types, as it does not represent feedback on past performance but rather feedback on planned action and anticipated outcome of that, i.e. *“I have got this situation and I was going to do this, is that the right thing to do?”* (Paramedic, P3906).

Debriefings were repeatedly mentioned as occurring after unusual incidents or those involving multiple resources, for example *“a serious trauma job, there will be multiple team members there and you will do a debrief after the job”* (ECA, P3916). Lastly, investigations and feedback from coroners’ reports were identified but mentioned least frequently by participants.

### Motives for seeking feedback

Upon identifying the different types of feedback mentioned by participants, it became clear that sometimes feedback was simply provided routinely as part of performance appraisals, but on other occasions was sought intentionally by EMS professionals. When actively seeking feedback, interpretation of the data revealed a wide variety of motives. The most commonly mentioned motive was to improve patient care, which was solely mentioned in relation to patient outcome feedback and was about the desire to *“improve the standard and quality of the care that I can provide as a clinician”* (Specialist Paramedic, P3904).

A desire for closure was a motivating factor for seeking several types of feedback, with a specialist paramedic (P3935) describing that patient outcome feedback *“help [s] you to put it to bed a bit more”*. A motive relating to all feedback types was desiring reassurance and praise with an ECA (P3910) stating that *“everybody wants to be told they’ve done a good job”*.

Another frequently mentioned motive was clinical curiosity or reducing uncertainty: *“Sometimes I would like to know that bit more, not the full ins and outs but to actually know whether or not I had gone down the correct pathway, or my hunch was right with what that patient was, the injury or what it was and if it wasn’t and I had missed something then I would like to know, so that I can learn from it”* (EMT, P3911). Clinical curiosity appeared to be heightened given the isolated working environment of EMS professionals, with one clinical supervisor (P3918) stating *“because they are autonomously treating and assessing patients they do need to know if they have gone the right way”*.

### Feedback mechanisms and outcomes

Basic themes related to participants’ perceptions of the outcomes of feedback predominantly addressed the learning processes of both professional and personal development. In the context of professional development, increased knowledge and reflection were perceived to lead to better decision-making and behavior change: *“You would know that you were doing it right or if you were doing it wrong, then you would change something”* (Paramedic, P3938) and *“It is for your own clinical development. It kind of builds your knowledge”* (Paramedic, P3905).

Another participant referred to the mechanisms of learning and reflection as *“recruiting their own clinical practices”* (Specialist paramedic, P3904) as exemplified in the following: *“It is nice to know [what happened to the patient] but also it just helps you to learn, to recognize symptoms. You know, if you’re thinking ‘it’s this’ and it turns out to be something else. You can try to recognize little pointers, little signs and symptoms that’ll help you make your decision next time”* (Paramedic, P3919).

Increased confidence was also perceived to play a role in professional development with one ECA (P3016) indicating that positive feedback *“gives you a bit of a confidence boost that you are knowledgeable in what you are doing”.* Similarly, there was an acknowledgement of the benefits of increased intra-professional dialogue: *“there can be a million ways in which you can do the same job, so it is quite good to get feedback from people. To get a pool of ideas together just for your own practice really.”* (ECA, P3916).

Concerning personal development, participants talked about the role of feedback in increasing staff mental health, job satisfaction and dialogue with peers: *“If you had a little feedback that ‘Actually you’ve done that really well’, ‘you made this sort of positive impact’, maybe it would have a little bit more impact on your wellbeing and it would sort of affirm in your mind that you’ve done the best that you could do. It would make everyone feel a bit better”* (EMT, P3926).

In many cases, participants related this back to their work and increased job satisfaction, with one EMT (P3925) surmising that *“getting a thank you would probably change staff’s mind set on things, willing to do more as opposed to digging their heels in the ground which a lot of us do because we’ve just had enough of being abused for such a long time”*.

These mechanisms and outcomes appeared to relate to the majority of feedback types and motives mentioned, although participants did not seem to link any of these outcomes to unsought feedback from routine appraisals, as illustrated in the arrows in Fig. 1.

## Discussion

This study aimed to explore the experience of EMS professionals regarding current feedback provision and their views on how feedback impacts on patient safety, staff wellbeing and professional development. Qualitative analysis of interview transcripts yielded rich insights into current feedback provision, feedback types, and motives for seeking feedback, as well as mechanisms and outcomes of prehospital feedback (Fig. 1). An important finding from this study is that EMS personnel viewed current feedback provision as inadequate and consistently expressed a desire for more feedback, especially those in a non-specialist, post-training role. Enhancing feedback provision was thought to improve patient safety by supporting professional development and clinical decision-making, facilitating reflection, knowledge acquisition and professional behavior change. Similarly, participants thought that enhanced feedback could improve staff wellbeing by enabling closure and encouraging intra-professional dialogue and peer-support.

An important contribution of our study to the existing literature is the identification of a potential typology of feedback for prehospital clinicians that includes: patient outcome feedback, patient-experience feedback, peer-to-peer feedback, performance feedback, feedforward: on-scene advice, debriefing and investigations or coroners’ reports. Although this typology is based upon testimony from one EMS context, it may serve as a useful model for further development of prehospital feedback interventions or further evaluative research. The reported inadequacy in current feedback provision observed in this study is congruent with other studies’ findings; although, all participants in this study were able to describe examples of when they had received feedback [7, 8, 31]. Study participants named several barriers such as patient confidentiality and the time and resources involved as possible reasons for inadequate feedback provision, consistent with those reported in the literature [44].

Prior audit and feedback research has identified a range of characteristics for effective feedback in non-EMS settings [12, 45, 46]. Our study provides insight into the characteristics that are important for feedback in prehospital care, including: timeliness, inclusion of explicit targets and individualized reports with relevant detail. The concept of providing feedback on ‘the right cases’ was similarly discussed in a smaller qualitative prehospital study where participants requested feedback on patient presentations with diagnostic uncertainty, incident closure, non-conveyance and patients discharged from ED without treatment [31]. These categories emphasize the increased complexity of decisions made by EMS professionals whilst faced with inadequate feedback provision, referred to as the ‘feedback paradox’ in other studies [47].

Although, FSB has been alluded to in other prehospital feedback studies [7, 31], we have sought to develop further insight through our analysis by identifying FSB motives. With the exception of improving patient care, participants’ motives for seeking feedback appear to fit within models used in the broader FSB literature [26]. The feedback-seeking motive of improving patient care was the only outward-facing motive, whilst clinical curiosity, desiring reassurance and praise and closure were inward-facing motives relating to self [26]. Interestingly, improving patient care was only mentioned in connection with patient outcome feedback, whilst patient-experience feedback was perceived as only being associated with desiring reassurance and praise. In contrast, the other types of feedback provided by hospitals, peers or EMS organizations were frequently mentioned in relation to clinical curiosity and closure (Fig. 1).

**Fig. 2:**
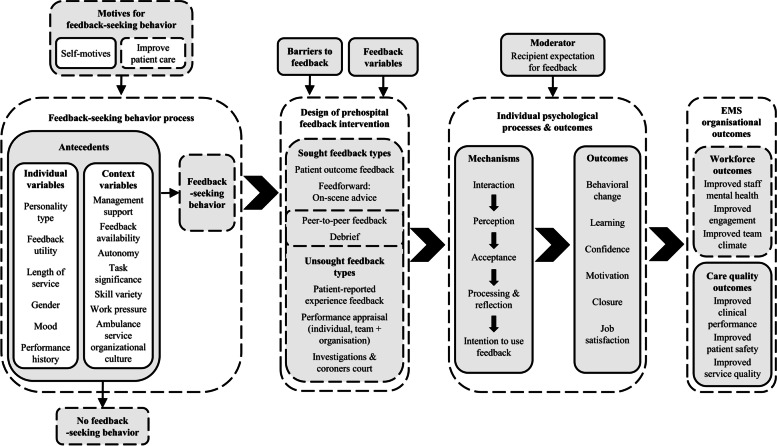
Logic model for prehospital feedback interventions

To consolidate the hypotheses and practical insights for future feedback interventions generated from our analysis, we developed a logic model combining themes from our analysis (Fig. 1), Brown’s CP-FIT model and FSB theory. The resulting framework is intended to serve as a guide for future research and intervention. Crucially, CP-FIT excludes sought feedback whereas our study suggested that participants viewed existing prehospital feedback as isolated occurrences, relating to individual patients and often involving feedback-seeking behavior [22]. Existing models from the FSB literature do not capture unsought feedback types or organizational outcomes described by our participants. We therefore combined elements from both theories in our logic model to account for the study findings concerning prehospital feedback (Fig. 2) [24, 25].

Most elements of our study findings were easily reconciled to the theoretical models. However, feedback-provider aspects could not be integrated as the interview study did not set out to explore this aspect of feedback provision. The new concepts generated in this study are: the typology of prehospital feedback; the seeking or non-seeking behavior; the motive of improving patient care and the outcome of improved team climate.

Mechanisms were difficult to separate from outcomes in our data analysis, which mirrors the poor understanding of feedback mechanisms reported in the wider health services literature [23]. One of our basic themes – reflection – has previously been mentioned as a feedback effect in the prehospital literature [31, 48]. However, viewed through the lens of CP-FIT we interpret reflection as a mechanism underpinning the effect of prehospital feedback [22]. Further research is needed to explore reflection as a mechanism within prehospital feedback and feedback mechanisms more broadly.

### Limitations

The study participants were professionals from the same EMS. It is also possible they had a particular interest in the topic. Therefore, direct transferability of certain contextual and perceptual observations concerning experienced feedback to a wider EMS population may be limited [49, 50]. The semi-structured nature of this qualitative study, however, permitted depth of insight into prehospital feedback processes and outcomes and the resulting logic model can be used to test transferability to other contexts.

Some interviews were undertaken by telephone which deprives the researcher of the usual non-verbal cues present in face-to-face dialogue, requiring greater reflexivity on the part of the researcher [51]. The analysis did not reveal any differences depending on how the interview was conducted, however, and the option of telephone interviews aided recruitment.

Participants often recalled examples of when they received or sought feedback, which may have been subject to recall bias. Future studies might employ different or complementary designs in which real-time data is collected when EMS professionals actually desire or receive feedback.

The lead author (CW) is a practising paramedic in a different UK ambulance service with her own clinical and feedback experiences, which might have influenced interpretation of the data. CW was not involved in data collection and arguably the researcher’s insider positionality facilitated a deeper insight into the data, supported by frequent discussions with non-clinical members of the research team (AH, JB) regarding decisions made during data analysis [52].

As previously described, abductive reasoning was employed when developing the logic model; therefore, we acknowledge that this model is provisional, tentative and in need of confirmation in keeping with the methodology we employed [37, 40]. We propose this could be done both by testing interventions based upon the model and further non-interventional studies to explore specific hypothesised mechanisms. In particular, exploring the effect of individual-level characteristics on impact and behaviors linked to feedback may be an important area of future study.

### Implications for practice and research

The effects of prehospital feedback are receiving growing attention in published literature [7, 31, 44]. Our study emphasizes the potential impact of effective feedback interventions on increasing intra-professional communication; which elsewhere has been identified as a cornerstone of complex prehospital decision-making [48]. The psychological impact of prehospital feedback has previously been highlighted in two qualitative studies, which similarly suggested that EMS professionals believe feedback has a positive impact on patient care [7, 31]. However, a recent literature review on prehospital feedback concluded that few interventional studies have been able to demonstrate any effects of prehospital feedback on patient outcomes [44].

Whilst our study was non-interventional, the proposed logic model has implications for current prehospital feedback practice and research, as EMS agencies could use it to design, monitor and evaluate prehospital feedback initiatives. Further research could include empirically testing our logic model in the prehospital setting or adapting it for use in other healthcare settings. Seeing how our logic model could be implemented to support professional development and performance management is an important future area to explore. With improved feedback systems in place, EMS professionals should be able to demonstrate that they are actively seeking and using feedback to support their professional development, which is a fundamental part of professional competency frameworks for EMS professionals [53]. In other specialties, such as anesthesia, strengthening performance feedback systems has supported professional validation and continuous improvement [54].

## Conclusions

This study emphasizes limitations in current feedback provision for prehospital clinicians and highlights EMS professionals’ strong desire for feedback. It builds upon existing studies by outlining a typology of prehospital feedback by source, identifying motives for seeking prehospital feedback and proposing a logic model that integrates current theory related to feedback in a clinical setting with insights from empirical data in order to guide future prehospital feedback interventions.

## Supplementary Information

**Additional file 1.** Interview guide.

**Additional file 2.** Additional quotes to illustrate themes.

## Data Availability

The datasets generated and analyzed during the current study are not publically available as sharing entire transcripts or long excerpts would violate the agreement to which participants consented; however, an extensive list of quotes is provided within the manuscript and supporting information files, and the datasets are available from the corresponding author on reasonable request.

## Abbreviations

CP-FIT: Clinical Performance Feedback Intervention Theory
ECA: Emergency care assistant
EMS: Emergency medical services
EMT: Emergency medical technician
ED: Emergency department
FSB: Feedback-seeking behavior
UK: United Kingdom
