# Supplementary material for: The role of feedback in emergency ambulance services: a qualitative interview study
Source: BMC Health Serv Res. 2022 Mar 3;22:296. doi: 10.1186/s12913-022-07676-1 (PMC8896262; doi:10.1186/s12913-022-07676-1)
Supplement: Supplementary file 1 — Additional file 1. Interview guide. [file 12913_2022_7676_MOESM1_ESM.docx]

**Additional file 1. Interview guide.**

**SECTION A: Introductory questions**

*Read standard statement concerning the purpose of the interview; take consent; explain confidentiality protocol; explain that interviewees are free to leave questions they don’t want to answer and end the interview at any point; gain permission to record; start tape, give researcher name, date and interviewee code; begin interview*

- How long have you been in your current role and could you briefly describe your professional background?
- Please could you tell me what your current role entails?
- What drew you to being a … [job role]

**SECTION B: Work-related determinants of wellbeing, engagement and patient safety**

*Wellbeing*

- How does your role and the work you do influence your wellbeing?
- What is it specifically about your day-to-day job that influences your wellbeing?
- How similar is this for your colleagues more generally?
- What are the main factors that affect the work-related wellbeing of ambulance staff in general? *(see list for prompts)*

*Engagement*

- What aspects of your job do you find particularly engaging?
- What aspects do you find particularly unfulfilling or frustrating? *(prompts)*
- How engaged is the workforce generally do you think in your organisation/profession?
- What changes to your role or the way you are required to do your job have helped/hindered your engagement in your role and/or your wellbeing? *(prompts)*

*Patient safety*

- What does patient safety mean to you in your day-to-day role?
- Is there a relationship between staff well-being/engagement (as you’ve described it above) and patient safety/service effectiveness? What is it? Can you expand?
- How have recent changes to the role influenced your ability to deliver safe care? *(prompts)*

*General*

- Are there any other aspects of the role that you think are relevant to wellbeing, engagement and patient safety that we haven’t discussed? *(prompts)*

**SECTION C: The role of feedback and follow-up in ambulance services**

- What is your experience of receiving feedback?
- What feedback do you currently receive?
- What effect does feedback have on your ability to carry out your role?
- How true do you think that is for your profession more broadly?
- What feedback would you like to receive?
- Do you ever seek feedback on what happened to patients you’ve dealt with? How? Why is this important to you? How might you use patient outcome feedback if more of it was available?
- *Probe for characteristics of desirable feedback (see list for prompts)*

**SECTION D: Existing and future support for work-related well-being**

- If you could change an aspect of the job to enhance your work-related wellbeing and/or engagement in your role, what would it be and how?
- How would you describe existing wellbeing and support systems for ambulance service staff at your organisation?
- What would you like to see done differently and how?

**SECTION E: Interview close**

- Is there anything else you would like to say before we end our time together?
- Thank you for completing the interview
- Give info/refer participant to PIS for what to do if they have any queries afterwards/how to contact the research team if needed
- Outline plans to feed back the results of the research to staff

**Prompts for work-related determinants which may impact on staff engagement, wellbeing and patient safety:**

- Individual factors – psychological and physical risk; experience in the role over time
- Team factors – “team” sense and conducive workplace culture; peer/social support
- Nature of paramedic work and job design factors: traumatic experiences (single events and longitudinal build-up); changes in the role; shift-working; individual working; time and resource pressures (lack of downtime/recovery time); diagnostic reasoning in the field; conveyance/non-conveyance decision making; risk to the patient in low-mid acuity cases; interdisciplinary collaboration (e.g. call-center; ED)
- Organisational factors – design of systems, processes and technologies; support from managers and the broader organisation in/out of the role; communication and quality of information in role; clinical supervision and opportunity for debriefing; feedback and follow-up on cases

**Prompts for feedback section:**

- Effects of feedback: wellbeing, personal professional development and learning, patient safety
- Purpose of feedback: professional development, enhanced wellbeing, patient safety – how might you use the feedback?
- Information content (focus and type e.g. data vs case narrative)
- Tone of content (focus and framing of messages, etc)
- Level of feedback/origin (on decisions you’ve made in the field/cases assigned to you or on quality and safety of the service; on conveyance or non-conveyance cases)
- Method/mechanism of feedback: How would you like to receive feedback?
  - Electronically by app/email/automated push delivery system?
  - Provision of general information that can be accessed by paramedics at base, for example
  - Debriefing by a colleague/clinical supervisor/manager/colleague in another area of the service
  - Time and opportunity to meet with colleagues to discuss data on service effectiveness and patient safety
- Who do you think should receive feedback? (question for service managers)
