# Supplementary material for: The role of feedback in emergency ambulance services: a qualitative interview study
Source: BMC Health Serv Res. 2022 Mar 3;22:296. doi: 10.1186/s12913-022-07676-1 (PMC8896262; doi:10.1186/s12913-022-07676-1)
Supplement: Supplementary file 2 — Additional file 2. Additional quotes to illustrate themes. [file 12913_2022_7676_MOESM2_ESM.docx]

**Additional file 2. Additional quotes to illustrate themes.**

| **Theme** | **Illustrative quotes** |
| --- | --- |
| **Feedback Provision** | |
| Current feedback provision | “If you want to get feedback on a patient that you’ve conveyed to the emergency department, you sort of have to go yourself and ask a doctor or ask a nurse about them” (Paramedic, P3912)  “*Do you receive any feedback*?” – “(sighs) No. No” (Clinical Supervisor, P3922)  “Feedback I receive? We don’t get any.” (Paramedic, P3937) |
| Desire for feedback | “It would be nice to know what happens with patients sometimes because when we drop them off at hospital, we don’t often know what happens to them […] Just to be able to find out what became of them.” (Paramedic, P3919)  “Yes, yes, [I would prefer to get that feedback] because sometimes I think the not knowing is kind of worse.” (ECA, P3916) |
| Barriers to feedback | “A lot of the time it could be because of confidentiality and people don’t want to give you information over the phone, there is no kind of formal method to get that information.” (Specialist Paramedic, P3904)  “It is hard because there is patient confidentiality and there is no form of mechanism so you are kind of relying on A&E hospital staff who are aware that you are the paramedic who treated ‘that’ patient and they know that and then they can join the dots and they are happy to pass that information to you.” (Clinical supervisor, P3922) |
| Feedback characteristics | “If I was always getting feedback and it was ‘Thank you, well done’, I think there would be a possibility you could become quite blasé about it.” (Paramedic, P3937)  “I just think something more structured somehow but I don’t know how that would happen in such an organisation like this” (ECA, P3910) |
| Antecedents | “I think some people in the ambulance service would be hesitant to engage with the process [of receiving feedback] because they’d feel that having a discussion with a more senior clinician is normally obviously disciplinary, rather than a sort of a learning experience or a ‘Oh actually well done, yeah, you did this really really good thing’” (Paramedic, P3912) |
| **Types of Prehospital Feedback** | |
| Patient outcome feedback (Hospital) | “A lot of the time there is not much follow-up from patients that you take to hospital. […] So, you take a patient to hospital and you don’t really find out what’s happened afterwards.” (Specialist paramedic, P3904)  “Sometimes I ask at the hospital if they can look up and see if the patient is alright. I don’t know whether we are supposed to but sometimes, when you get a patient in and you are not sure, some affect you more than others. You get quite close to some patients and you want to know what has happened. It is a big frustrating part of the job.” (Paramedic, P3906) |
| Patient-reported experience feedback (Patients) | “You can get feedback from patients. They can send in compliment letter and things and it is always quite motivating to receive.” (ECA, P3916) |
| Peer-to-peer feedback (Peers) | “But then the other feedback you do get, could be you’ve just done a job and at the end of the job it might be your own crew mate that says: ‘Do you know what you did really good on that job’.” (ECA, P3910) |
| Performance appraisal (EMS organization) | “We do development reviews yearly with the manager or supervisor and you can go over what you want to do, how you want to progress, it is more about your own career really.” (EMT, P3928) |
| Feedforward: On-scene advice (EMS organization) | “Sometimes you might be alone and you’re not sure what the best treatment is for this patient. You can always ring the clinical hub and they can give you some advice.” (Paramedic, P3905) |
| Debriefings (EMS organization) | “My experience with feedback is: you know if you’ve done any major jobs, you get some debrief and personally, if I need to discuss things I’ll ring the clinical supervisor or the clinical hub.” (Paramedic, P3905)  “We can get feedback from supervisors, obviously when they have been out with us but if you have been on a job, something like a serious trauma, there will be like multiple team members there and you will do like a de-brief after the job. So, you will get feedback on how you did and that is really good moving forward or you know new things to learn like what you could have done differently, what you could have done better or what you think went well.” (ECA, P3916) |
| Investigations & coroners court (EMS organization) | “Some of the big jobs that the police are involved with and you go off to coroners court, you might get a little bit of feedback from there.” (EMT, P3926) |
| **Motives for Seeking Feedback** | |
| Improve patient care | “When it [feedback] is related to clinical care it definitely improves the standard and quality of the care that I can provide as a clinician, especially if you are learning from other people challenges, or things you found difficult, or things that haven’t gone as well. It makes you more aware so that you if you encounter those sorts of problems you are kind of better prepared to deal with them and manage them.” (Specialist paramedic, P3904)  “I can use that feedback as part of my reflexive process so it contributes towards my learning. Feedback is always good because, like I said, I’m always learning. I’d ask them: ‘Would you do anything differently?’. So I’d use it ultimately as a self-development tool but also to then improve patient care because everything I do to develop myself is going to ultimately benefit the patient and the service that they use. So if more feedback was there then it would be very useful.” (Paramedic, P3932) |
| Clinical curiosity | “You want to know if somebody is alive because it’s hard to carry on when you’ve invested your time with somebody, to not know whether they’re alive or not. That’s hard.” (Specialist paramedic, P3930)  “I think for me it [wanting feedback] is just out of interest. I don’t know if it would affect the way I practice because at the moment not knowing is just part of my job, you just do your best every day.” (EMT, P3928)  “So you take a patient to hospital and you don’t really find out what happened afterwards? From a learning point of view that is really difficult. You question yourself ‘Have I made the right decision?’ or ‘Was that the right thing to do in this situation?’ or ‘What happened to that person?”. You might have seen a [certain] side to it and you think ‘Was that a such and such, or was that something else?’ ‘Have I made the right diagnosis?’, ‘Did I make the right decision to give these drugs or not?’. Things like that. Or even ‘Did I do the right thing taking this patient to hospital or not taking this patient to hospital and referring to a community service or different pathway?’ There is no feedback about those decisions.” (Specialist paramedic, P3904)  “Because they are autonomously treating and assessing patients they do need to know if they have gone the right way.” (Clinical supervisor, P3918)  “A lot of the time we are on our own on the road. You are kind of your own boss. So, because they [senior managers] are not seeing you actually do your job it is hard for them to give feedback.” (Paramedic, P3919) |
|  | “Feedback is the most crucial thing and I know it would help certain people. Because, I have seen paramedics who aren’t good at certain jobs, who have gone to that job, like say a trauma job. And I know a certain paramedic who went to a trauma job and she doesn’t really do trauma and she was in pieces after the job because she didn’t know if she had done right or wrong. There was nobody there to say ‘Yes that was alright that’, there was nobody there to say that.” (Paramedic, P3924)  “You know I want to know, I want to learn, and if I’m not doing something right I want somebody to tell me because otherwise I don’t know and I’m going to keep doing it wrong.” (ECA, P3910)  “Sometimes I would like to know that bit more, not the full ins and outs but to actually know whether or not I had gone down the correct pathway, or my hunch was right with what that patient was, the injury or what it was and if it wasn’t and I had missed something then I would like to know, so that I can learn from it.” (EMT, P3911) |
| Desiring reassurance and praise | “Everybody wants to be told they’ve done a good job” (ECA, P3910)  “Just a bit of acknowledgement of ‘well done, you have done a good job there’, To realise ‘Yes, I know my stuff, I have put my skills to work’, I think is something positive, definitely. I think it would put a bit more of a spring in my step of the reason why I have done all this training, the reason why I have re-read a book or gone on system training in my own time. I have made a positive impact.” (EMT, P3911)  “I think more patient feedback is good because I know I can speak for many people, when we get a compliment letter it makes you feel really good, it makes you feel motivated at work and we don’t really get them often enough just because of the way of inputting it, but I know when anyone gets that you always chat about it and you know it is a confidence boost for you really, so I think that is the most important” (ECA, P3916)  “I think it [lack of feedback] does lead to some frustration because you certainly don’t know if what you’re doing is the right thing and actually if it does have any effect. So, then there’s maybe the concern that you’re not doing the right thing or actually why are you bothering sometimes.” (Paramedic, P3912) |
| Closure | “There are patients I still wonder about now and again reflectively after many years I think to myself were I just starting my career now or giving someone advice I would say that anything that feels significant to you try and find out what happened and because it might help you to put it to bed a bit more.[…] I think it is a shame that I sometimes pass an address now or addresses and think ‘I wonder what happened to that person?’, ‘I wonder if they survived?’ or ‘did that person ever go home?’. I guess if you know the outcome sometimes it means that you just don’t ever think about them again and that is a good thing.” (Specialist paramedic, P3935)  “Going to hospital and just asking what happened to patient, sometimes that gives you a little bit of closure.” (Paramedic, P3919)  “Sometimes if you get a bad job, you try not to dwell on it, you walk away and you think ‘That job is done’. I am pretty good at doing that actually, at saying ‘That was a bad job but it’s done now’, I need to move onto the next one.” (Paramedic, P3906) |
| **Feedback Mechanisms and Outcomes** | |
| Increasing knowledge | “It is for your own clinical development. It kind of builds your knowledge.” (Paramedic, P3905)  “It is nice to know [what happened to the patient] but also it just helps you to learn, to recognise symptoms. You know, if you’re thinking ‘it’s this’ and it turns out to be something else. You can try to recognise little pointers, little signs and symptoms that’ll help you make your decision next time.” (Paramedic, P3919)  “We kind of sometimes ask the clerk at the hospital how a patient is or what happened, or I do anyway now and again. It is just for my own learning, so if I go to similar things in the future, I will be able to use that information.” (EMT, P3928)  “It [feedback] would definitely improve peoples’ learning and their ability to recruit their own clinical practices.” (Specialist paramedic, P3904) |
| Increasing confidence | “I’m not a very confident person so I like to know that I’m doing things right. It [more feedback] would make me feel better.” (Paramedic, P3938)  “Maybe you are going along the lines with a job and you are thinking what the diagnosis could be and then you get to the hospital and you check up on your patient and you will have done something right. It just gives you a bit of a confidence boost that you are knowledgeable in what you are doing. Or maybe if you get good feedback from patients then they can send in compliment letters and that is always quite motivating to receive.” (ECA, P3916)  “To be told that what you have done is correct and has worked, you might be able to use that in the future, it might help your confidence as well.” (EMT, 3928) |
| Reflection | “It is just about self-reflection, to make sure that I have not missed anything. So that I have learned going forwards and if I had a similar job like that again, I wouldn’t miss anything.” (EMT, P3911)  “I have just dealt with a patient this morning who has gone to Leeds following a road traffic accident. Within the next sort of two weeks I will get a detailed e-mail back from our trauma co-ordinator detailing exactly what injuries the patient sustained if any, so you can sort of reflect on your assessment and your treatment regime as a result of that, which is obviously really good.” (Clinical supervisor, P3918)  “It [feedback] helps you reflect on your practice doesn’t it? It helps you think: ‘Would I have done it the same way?”. If it is a positive outcome – well, not only if it is a positive outcome – if it is an outcome where you know everything was done, then that can be self-gratifying as well, can’t it? Because you think, well ok this person didn’t survive but actually knowing what happened and knowing that we got them there in time and they went down to surgery or this happened or that happened. Actually, this person had the best chance and that can be enough can’t it?” (Specialist paramedic, P3935) |
| Improving decision-making | “There’s a lot of jobs where you know little old people on the floor, you’re imagining, you know they’ve got a fractured hip but we never find out, we don’t find out if our clinical decision making was right if they have got a broken hip” (Specialist paramedic, P3930)  “You wonder what happened with that patient or what you could’ve done differently. So, if we did get feedback it would be quite important to us and we’d probably be able to do the role a lot better.” (ECA, P3927) |
| Changing behaviour | “You would know what’s happened to the patient but also you would get to learn sometimes if your initial thought was right or wrong. That allows you to alter your judgement next time.” (Paramedic, P3919)  “[When receiving feedback] you would know that you were doing it right or if you were doing it wrong, then you would change something.” (Paramedic, P3938) |
| Intra-professional dialogue | “There can be a million ways in which you can do the same job, so it is quite good to get feedback from people. To get a pool of ideas together just for your own practice really.” (ECA, P3916)  “Because quite often you can present at a job and it’s like ‘Woah I’ve no idea what’s going on here, but we’ll go down this line and we’ll treat for X, Y and Z’. So, if you get feedback you can then use it as learning and you can share things with other people as well and it’ll be like ‘I went to this job and it was this…’” (ECA, P3910) |
| Job satisfaction | “Positive feedback is always going to have a positive impact on my wellbeing. Going back to the job satisfaction you can think, you know I should be proud of that. Not being big headed, you can be proud of it, what you’ve done. And also when you find out about a patient that’s improved hopefully you can be happy they are improved and think ‘I’m less worried now’.” (Paramedic, P3932)  “Us getting a thank you would probably change staff’s mind set on things, willing to do more as opposed to digging their heels in the ground which a lot of us do because we’ve just had enough of being abused for such a long time” (EMT, P3925) |
| Staff mental health | “If you had a little feedback that ‘Actually you’ve done that really well’, ‘you made this sort of positive impact’, maybe it would have a little bit more impact on your wellbeing and it would sort of affirm in your mind that you’ve done the best that you could do. […] It would make everyone feel a bit better.” (EMT, P3926)  “It just feels like you’re always given to them [senior managers] and they never give anything to us. We don’t get any reward or any thanks. We just don’t feel appreciated or valued. It makes you feel rubbish. Because I spend all shift every shift doing the very best I can being nice to people, helping people. And it’s really nice when a patient thanks you or a family thanks you but you think ‘Oh, my managers could just say once in a while ‘You’ve done a really good job, well done.’’ But I’ve never once heard that. [*And if that changed and they did do that, how would that affect you?]* It wouldn’t change my work because I’d still do the best that I can but it would just make me feel more appreciated and make me feel better really. Like I am going to a nice place rather than a rubbish place just to get paid.” (Paramedic, P3938) |
